# American Architecture*The substance of a lecture, one in a course on Architecture, before the Literary Society of the First Presbyterian Church, in Elmira, last winter.

**Published:** 1883-07

**Authors:** Ausburn Towner


### * AMERICAN ARCHITECTURE.

AUSBURN TOWNER.

To say "American Architecture," is to use a very faulty combination of terms, if to the two we attach their conventional meanings. There is no such thing as American Architecture, as we speak of Chinese, Egyptian or Swiss Architecture, and in the sense of the style or art of building in America it means pretty nearly every kind of building known since the time of Jabal. Our country is original and unique in but one thing, its marvelous system of government. In every thing else, it has drawn from every nation on the face of the globe, and in no other direction has it depended more upon what has been done before by other races, than in architecture.

What *has been* the style of building in this country is a matter rather of curious interest than much value. Being merely the result of necessity, it has been careless, even slovenly, and has laid the foundation for nothing to come after, that is peculiar or distinctive.

There might something have grown out of the rather striking edifices that our Dutch ancestors built in New York, Albany and Schenectady, some of which are still standing. They have the elements of a certain picturesqueness about them that is pleasing amidst proper surroundings. But when Peter Stuyvesant with his wooden leg and his sturdy neighbors were driven away by the English, every thing that was Dutch, even to the names, disappeared, too, and exist now only in memory, in smoky paintings, or out-of-the-way, forgotten places.

There might something also have grown out of the primitive Indian wigwams and log houses, certainly peculiarly indigenous originals. From as humble beginnings came some of the gems of Architecture that the world admires. But the American Indian is about the only race from which America has borrowed nothing, unless it may be the land itself, and a homelier but stronger term is more appropriate to express how *that* was obtained.

There were possibilities in the pioneer's log cabin, that to a genius might have been suggestive of a style we could have claimed for our own. The original model of the Parthe-

* The substance of a lecture, one in a course on Architecture, before the Literary Society of the First Presbyterian Church, in Elmira, last winter.

non was of a ruder nature than most of the dwellings where our rugged first settlers lived while they were subduing the wilderness. But just as soon as the sharp teeth in the woodland saw mill went rasping through the logs, seeming to shriek in Satanic glee over what it was preparing, the comfortable hut fell rapidly into disuse and was forgotten in a frame house, prim, stiff and angular, of pretentious, New England meaningless design, staring brazenly out over the protesting stumps of fallen trees, with its most brilliant emerald green blinds and the shiniest white coat, like a ghost parading in green goggles!

There was such a great spread of land, even in the cities, that no study was required in building, certainly none in the direction of compression. When the decision to build was arrived at, the only question was the quantity of material to be obtained, and, that settled, work was begun. There were so few architects then, men who had made of the art a study and a life-work, that there could hardly be said to be any. The carpenter or master-builder was his own architect; the general plan, if it was a public building, being furnished by a committee who probably knew as much about architecture as they did about a transit of Venus; or if it was a dwelling, by the wife or mother of the owner of the property. In this way, up to within a score of years, many of the public buildings and most of the homes in this country, certainly in this city, were put up. It would not be surprising if some droll error had occurred under such circumstances. I know of a house in this city, that, when originally finished, possessed not one solitary closet; another, where a place for the stairs to the second story had been forgotten, and another where no provision had been made for a single chimney.

These architect-carpenters had one idea as to the general appearance of the building to be erected, whatever the interior was to be, and they clung to it tenaciously. You could, until within a few years, and can, even now, measureably identify all over our town, the hand-writing, so to speak, that certain of these builders have left. They might as well have traced their names and the year of their work over the doorways. One, during his active life, built or suggested all of those long and narrow candle-box dwellings, with their gable ends to the street that you could count so numerously on every thoroughfare in the town. Then came those somewhat castellated brick houses, one of which is so conspicuous on the Horseheads road, and another, a little beyond the southern end of the Lake street bridge, that bears cut in stone over its main doorway, the strange latin sentence: "*Auster portus diversoriun,*" supposed to mean "Southport Hotel." Then came the era of those semi-Greek houses, bearing those high and heavy columns on their fronts, for what purpose, who can tell? Three of which now grace, and at one time four in a row graced, what many consider to be the handsomest thoroughfare in town. Then followed those square, dry-goods-box-looking houses, the roof running to a point in the center, and sometimes surmounted by a cupola, with a little projection at one side, too small to be called a wing and too large for a bay window. It will be remembered by many of my older readers, that the three principal church buildings in town, were, at this time precisely alike as to their general architecture, in which they were joined by the county court house. Observing men in any community, will tell you very nearly, the date of the erection of any building that is more than a score of years old, simply by its general appearance.

Throughout the whole country, this duplication and reduplication of some prominent, but not distinctive or characteristic design or style was to be observed. The Boston State House had its counterpart in about every capital in the Union, and the City Hall in New York, with its mild imitation of the facade of St. Peters, at Rome, and souvenirs of a number of other conspicuous buildings in the old world, stood the constant model for other municipalities that could rise to the dignity of having buildings of their own. All the theatres, inside and outside were so like the "Old Park," that a close examination alone, would satisfy an inquiring stranger as to the place where he was. All college buildings were laid out in the same uniform parallelogram preciseness. Every church, nearly, in New York and our larger cities was but a copy of Trinity, which itself was a duplicate, in appearance, of the old "North Dutch," which eventually became the post-office of the city, that, too, being modeled from some home church, whose tall spire cast, and still casts its shadow in the Zuyder Zee.

What *has been* the architecture of America, can be easily summed up in the words: imitative, commonplace, pointless and without motive, save that of necessity, and, in most cases, absurd. In this art America is without a past.

What *is* the architecture of our country is a subject that opens a broader and more interesting field of inquiry, although it is but little more satisfactory to our patriotism. The art is beginning here to take the lead of all its sister arts The greatness of the country, overflowing State and National treasuries and the colossal fortunes amassed by many of our citizens, find readier and more manifest expression in this art than any other. While in this state of affairs lie the chances for its greatest development, in them also lies its greatest danger, that it may become vulgar, obtrusive, degraded, flaunting itself in excessive ornamentation; making cost stand for value.

The first characteristic of our architecture that would strike an unprejudiced, observing eye, would be the impression it seems designed or desirous of conveying, of—I was about to say vastness, but that term is too large for what I wish to describe, involving in it something of the expanse of the sea or the heavens. Massiveness will not do either, for that implies bulk and weight, and largeness means nothing at all. I will say bigness. This seems to be the first characteristic observable now in our architecture. A self-asserting bigness, not relatively so because something else is smaller, but big by itself. As though a prevailing sentiment of a country that had the biggest rivers, the biggest waterfalls, the biggest chain of lakes and the biggest gold mines in the world, was struggling also to find similar expression in its architecture. The first sentiment I have heard uttered by persons looking at the Capitol in Albany, or in Washington, certainly conspicuous and fair examples of American architecture, has often been: "Whew! What a big building!" No such notion ever entered the head of a person viewing for the first time, St. Peters at Rome, the Milan Cathedral, or the Pyramids, structures which are, indeed, immense.

Americans, I know, have always had a vivid sense of the extraordinary dimensions of their country and its institutions, for which they perhaps have good grounds, but it would seem that only since the war, after they had raised the biggest armies ever in the field, and fought some of the biggest battles known to history, that the notion seized upon them that their architecture must mutely express, in some degree, their idea of their size as a Nation. The main building of the centennial at Philadelphia will be remembered. It was one of the largest edifices ever put up under one roof. Look at those enormous blocks building in New York and other large cities, some for business purposes, others for apartments or flats, covering nearly whole squares and towering into the heavens ten and eleven stories in height. There is Powers's block in Rochester, the representative building of Western New York. It is a small mountain of stone, iron and mortar! The architecture of hotels has grown to such tremendous proportions that all of the principal buildings in the country devoted to such purposes deserve the epithet, mammoth. The halls in some of these caravanseries are so long that to see from one end to the other, requires a telescope of considerable power, and it is not much of an exaggeration to say that a bell-boy requires half-a-day for the journey from the office to the most remote room and back again.

This characteristic of size is by no means confined to buildings of a public character, of which examples could be given by the page, but is also to be seen in private residences. Think of a dining room for a private American citizen, fifty feet in length, thirty-five feet in width and thirty-two feet in height! Not an exceptional instance, for they can be counted by the score in most of the larger cities of our country. Invading our own town, the characteristic is to be met with in more than one instance in mansions that doubtless are a delight to the eye of the passer-by, but which, I venture to affirm, have often given rise to the expression of the careful and wearied wife and mother: "Oh! this house is too big!"

The next characteristic of our country's architecture is costliness, or at least the appearance of it. The elaboration of ornamentation from which this costliness arises, is so profuse, as to be, in many instances, excessive. There really seems no limit to the application of cut and carved stone to the exterior and interior of edifices going up in every section of our country, and if the attainment of this material is not possible, the daring ambition of the American equally sees no limit to the plastic qualities of stucco, pine boards and galvanized iron. It hardly seems necessary to cite instances where this characteristic is prominent, for they exist on all sides of us, and he who runs may read. If I again refer to the capitol at Albany, it is because it is near to us and makes an easily-perceived illustration. It is by no means the only one. No American has ever referred to it yet, without somewhere in the course of his remarks having said: "Why, it has already cost between twelve and thirteen millions of dollars, and is not yet finished!" Viewing the edifice with such sentiments and having a glimmer of an artistic impulse hidden somewhere in your organization, it is no satisfaction to add that the great Empire State is abundantly able to foot the bill. No treasury is deep enough to stand the drain of ill-directed extravagance.

Costliness is certainly a prominent characteristic of our American Architecture, when we have private dwellings, like one not twenty-five miles from Elmira, into whose dining-room, of only moderate dimensions, have been crowded $30,000 worth of ornamentation and display, and when in our chief cities from Boston to San Francisco, there are others, which with all of their appointments complete, absorb sums running up as high as $5,000,000. It may be said that these are exceptions. Granted that they are, but they attract wide-spread attention; are what give character to the whole country and are those things by which it is known and judged of in other lands and other times.

As to the characteristics of beauty and style, there is doubtless a striving after the first, but no conspicuous public example has been attained thus far, and as to the second, none predominates nor has any been originated.

These four named, characteristics, may be said to be the exterior or looked at side of architecture, and so, first attracting general observation. There is still the interior or 'lived in' side of the art that deserves attention, for in this our American Architecture seems to be taking the lead. This side comprises the characteristics of comfort, convenience and healthfulness. America may fail on the fine art side of Architecture, but in so far as it is a useful art, our country, in this as in many other, practical every-day directions, is the peer, if not the superior of most other nations. Only brief reference need be made to what all should be informed of. In all of these public and private buildings to which I have made some allusion, and in most others of any pretensions, the comfort of the occupant is enhanced in every possible direction. The flights of stairs are laid at a wide angle or the ascent and descent is quickened and made easy, by noiseless elevators that are constantly in motion, so that the building, in this respect, may be twelve or twenty stories in height, as well as two; heat is provided, its quantity regulated by a twist of the wrist or ankle, and light, when the natural supply fades, by a motion of the thumb and finger; water lifts itself at the beck of the hand to any altitude, and the system of ventilation has been worked out so perfectly, that the air can come as pure to the lungs in the most crowded neighborhood, as though it was breathed at the top of a hill in the country. These contrivances and others are not of exceptional appliance, public sentiment compelling their appearance in shops, hotels, school-houses, even prisons, wherever, in fact, there are likely to be gathered large bodies of individuals.

But these characteristics are to be seen in no greater perfection than in the multitude of unpretentious, but charming dwellings that are being erected in every corner of our country. They give one no idea of "bigness" nor costliness, and the style may not be classical nor even artistic, but comfort manifests itself in every line and proportion, while convenience is the element most apparent at every turn. Perhaps it is just as well for us not to emulate Egypt, Greece or Rome in the matter of temples or other public structures. You may speak of the land celebrated for its coliseum, its ducal palaces, Louvres, Alhambras, or wonderful mausoleums. They are but hard, cold, unimpulsive words at the best. But how the heart will warm and the affections turn to that country of which you can say, as can more and more truthfully every day be said of America: "It is a land of comfortable homes!" In future ages, which characteristic will be read of with most interest, or with the tenderest memories?

As to what has been and what is the architecture of America, there can hardly be any disagreement, but what American architecture is to be or may be, is something that it would be difficult to foretell. We have no ground on which to form a basis.

There is a conjecture or guess, hardly a prediction, more a hope founded on a dream, fostered by some of the thinkers, observers and philosophers of the old world, that out of the heterogeneous mass of people of this country, gathered as they are from every land and race on which the sun shines, there will, in time, be coalesced and united, a homogeneous people, peculiar and distinct, high in grade, mentally and physically, unlike any other yet known to history, with a civilization so refined that it will be without parallel. If time brings this result, it would be no more than just to expect that with it there will also come a literature distinct and peculiar, a school of painting of its own unique character, and an architecture suggestive to this land and absolutely entitled to its name.

But, without exaggeration, this is a vast country. The bracing air of Norway, the gentle temperature of the South of France, the sunny skies of Italy, the bleak hills of Scotland and the rugged shores of Greece, all have their counterparts and parallels within our borders. The people of the lands I have named, have no more diverse surroundings, with the single exception of political aspirations, than do the people of the various sections of our country, and it is the surroundings that give color, tone and direction to any mental leaning. Especially do they govern in an art like architecture, where foundations are so laid upon the useful and the needs of man. More than this, it is not probable that our country will ever be cut off from a constant and instant communication with the old world, and it is not possible that we will ever grow beyond the influence, in art, of the polished nations of ancient times or the cultivated ones of the age.

I know that the frequent and rapid means of communication between all parts of our country, and the constant interchange of sentiment and persons that are going on, tend largely to assimilate separate communities into something like a general likeness, and although there may, in literature and art, arise certain distinctive features in different localities, it is much to be doubted if there will ever be a style or manner of building in this country of such general acceptance, and so thoroughly without a counterpart, as to be entitled to the generic name of American Architecture.

A correspondent of the *British Medical Journal* states that he has found the application of a strong solution of chromic acid three or four times, by means of a camel's hair pencil, to be the most efficient and easy method of removing warts. They become black and soon fall off.

